# The Elements of Therapeutics

**Published:** 1878-01

**Authors:** 


The Elements of Therapeutics. A Clinical Guide to the Action of Medicines. By Dr. C. Binz, Professor of Pharmacology in the University of Bonn. Translated from the fifth German edition and edited with additions, in conformity with the British and American Pharmacopœias, by Edward I. Sparks, M.A., M.B., Oxon, member of the Royal College of Physicians, of London, etc., etc. New York, Wm. Wood & Co. 1878.

The book is made up of twelve chapters, each giving the action and uses of the remedies belonging to a particular class. The classes mentioned are: Nervous sedatives, nervous stimulants, ætherial oils, demulcents, astringents, plastics, diathetics, antiseptics, antipyretics, evacuants, cauterants and mechanical agents. Each particular remedy associated in the respective classes is not elaborately, though sufficiently, described, and the physiological action, dose, etc., given in regular order.

This is intended and answers for a text-book of materia medica and therapeutics. Although the classification does not strike us as the very best, yet so very much in advance of some others that we must advise its perusal by students and practitioners. The actions attributed to various agents are not in accordance with our opinion of them, yet as gross errors have been indulged for a century past in this particular, we are pleased to see a change, and shall not attempt their refutation.

